# A peculiar presentation of tamponade: pericardial mesothelioma

**DOI:** 10.1093/jscr/rjae279

**Published:** 2024-05-06

**Authors:** Syed Danial Syed Ahmad, Frazer Kirk, Wisalya Wijesinghe, Cheng He, Andrie Stroebel

**Affiliations:** Department of Cardiothoracic Surgery, Gold Coast University Hospital, Level 4, D Block, Gold Coast University Hospital, 1 Hospital Boulevard, Gold Coast 4215, Australia; Department of Cardiothoracic Surgery, Gold Coast University Hospital, Level 4, D Block, Gold Coast University Hospital, 1 Hospital Boulevard, Gold Coast 4215, Australia; Bond University Faculty of Health Sciences & Medicine, 14 University Dr, Robina QLD 4226, Australia; Department of Cardiothoracic Surgery, Gold Coast University Hospital, Level 4, D Block, Gold Coast University Hospital, 1 Hospital Boulevard, Gold Coast 4215, Australia; Department of Cardiothoracic Surgery, Gold Coast University Hospital, Level 4, D Block, Gold Coast University Hospital, 1 Hospital Boulevard, Gold Coast 4215, Australia

**Keywords:** pericardial disease, mesothelioma, pericardial mesothelioma, rare cardiac disease, rare cardiac case, surgical dilemma, pericardial tamponade

## Abstract

Pericardial mesothelioma (PM) is rare with only 200 cases recorded, and a post-mortem prevalence of <0.0022%. It is the third most common cardiac/pericardial tumour, behind angiosarcoma and rhabdomyosarcoma. PM incidence increases with age, typically incidentally diagnosed between 50 and 70 years, with a 3:1 male predominance. Occasional PM can cause chest pain, dyspnoea, cough and even dysphagia. PMs are often misdiagnosed with only 25% of cases being antemortem diagnoses. Unlike pleural mesothelioma, the link between asbestos exposure and malignancy is less convincing, with only 20% of cases having known exposure. 6 There are three histological types: epithelioid, fibrous (spindle cell), and biphasic (mixed). The average life-expectancy post diagnosis is 3–10 months. Due to the heterogeneity of the presentation and rarity there is no standardized management algorithm, and the diagnostic imaging or laboratory investigations are scarcely described. We are presenting one of the cases diagnosed in our unit here in the Gold Coast.

## Introduction

Pericardial mesothelioma (PM) is a rare with only 200 cases recorded, and a post-mortem prevalence of <0.0022%. It is the third most common cardiac/pericardial tumour, behind angiosarcoma and rhabdomyosarcoma [1]. PM incidence increases with age, typically incidentally diagnosed between 50 and 70 years, with a 3:1 male predominance [2]. Occasional PM can cause chest pain, dyspnoea, cough, and even dysphagia. PMs are often misdiagnosed as constrictive pericarditis, pericardial effusion, cardiac tamponade, and congestive heart failure, and >50% of cases present with metastatic disease, to the liver, lungs/pleura or kidneys. Only 25% of cases are antemortem diagnoses. Unlike pleural mesothelioma, the link between asbestos exposure and malignancy is less convincing, with only 20% of cases having known exposure [3]. ^6^ There are three histological types: epithelioid, fibrous (spindle cell), and biphasic (mixed) [3]. The average life-expectancy post diagnosis is 3–10 months [4].

Due to the heterogeneity of the presentation and rarity there is no standardized management algorithm, and the diagnostic imaging or laboratory investigations are scarcely described.

## Case summary

A 62-year-old female presented with increasing cough, orthopnoea and NHYA Class II dyspnoea. Associated with post-prandial right upper quadrant abdominal pain. Without significant medical history. Examination revealed mild, Murphy’s negative right upper quadrant tenderness and biochemistry demonstrated mild liver function derangement.

Abdominal ultrasonography was negative for biliary pathology. She was treated empirically for a respiratory tract infection due to the presence of cough and dyspnoea.

One week later she represented to hospital with NYHA Class IV dyspnoea and severe reduction in exercise tolerance to 100 m. Pulmonary embolism and pericarditis were excluded with a negative d-dimer. Full blood count, biochemistry and electrocardiogram were unremarkable.

A Chest X-Ray (CXR) revealed an enlarged cardiac silhouette. Trans-thoracic echocardiography (TTE) confirmed a large pericardial effusion.

**Figure 1:**
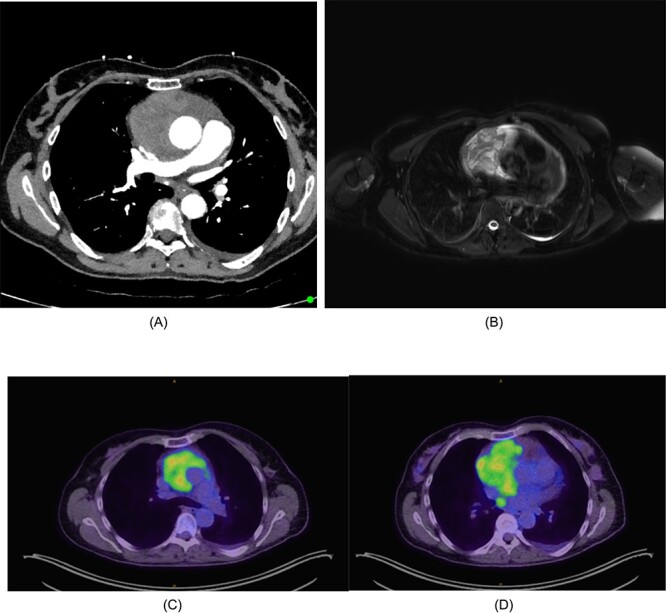
(A) CT chest/abdomen/pelvis demonstrating pericardial mass. (B) Cardiac MRI displaying cystic mass in the pericardium with small amount of pericardial fluid surrounding the lesion. (C) FDG-PET from the vertex to the upper thighs illustrating infiltrative lesion centred within the mediastinum. Small left pleural effusion noted. (D) Further focus of activity within the posterior mediastinum in the right para-oesophageal region.

Pericardiocentesis was performed draining 2.5 L of fluid. However, the enlarged silhouette remained on CXR. Cytology of the pericardial fluid demonstrated scant atypical mesothelial cells. Persisting dyspnoea and development of pre-syncopal symptoms despite pericardiocentesis prompted a computer tomography (CT) scan, which demonstrated a large mediastinal mass encasing the aorta (Fig. 1A).

The mass had a solid-cystic appearance on cardiac magnetic resonance imaging (CMRI) (Fig. 1B) encasing the aorta circumferentially and occupying the anterior and middle mediastinum. This lesion was intensely FDG-Avid on positron emission tomography (PET), with an isolated right para-oesophageal nodal metastasis (Fig. 1C and D).

**Figure 2:**
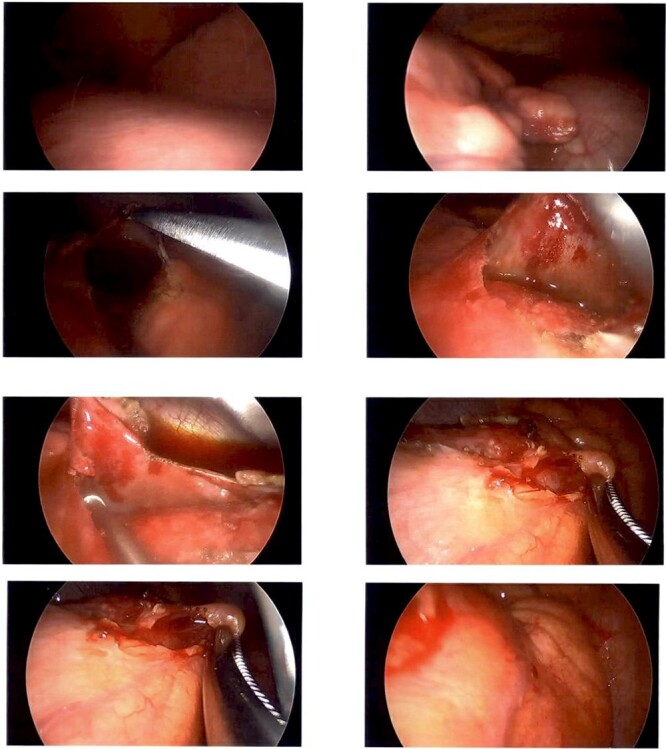
Intraoperative photography from left sided VATs biopsy. Showing a firm cream-coloured nodular and globular intra-pericardial mass containing gelatinous fluid was found circumferentially wrapping the ascending aorta.

A CT guided biopsy was preformed to attain a diagnosis, which was insufficient for diagnostic purposes. Subsequently a left video-assisted thoracoscopic surgical (VATS) biopsy (Fig. 2), was performed which again was non-diagnostic.

A Hemi-sternotomy was performed to re-sample the lesion. A firm cream-coloured nodular and globular intra-pericardial mass containing gelatinous fluid was found circumferentially wrapping the ascending aorta. Thymic tissue looked grossly normal.

Post-operatively the pericardial effusion recured and started to produce echocardiographic signs of tamponade. A pericardial window was fashioned via a left VATS approach, to prevent further tamponade.

Histological analysis showed pericardium infiltrated by epithelioid tumour papillary structures invading the stroma and lymphatic tissue within the mass. Immunohistochemistry was positive for mesothelial markers of calretinin, CK5/6, BAP1 and WT1 and negative for Desmin and TTF-1. Consistent with a pericardial mesothelioma of the epithelioid type (T4N1M1) and referred to medical oncology.

## Discussion

Retrospectively this presentation is explained by the pericardial effusion the mesothelioma produced, however give the exceeding rarity of the condition it was understandably not considered initially. The abdominal pain and liver function derangement may have been an early sign of hepatic congestion due to tamponade. Given the lack of constitutional symptoms, absence of smoking or asbestos exposure, other differentials including Castleman’s disease, Thymoma and Lymphangioblastoma were considered in the Thoracic Oncology multidisciplinary team meeting, however the intense FDG-Avidity was highly suspicious for malignancy.

Classic of mesothelioma, three tissue/fluid samples were inconclusive for diagnosis, but all suggested mesothelial cell origin for the lesion, which has been recorded in up to 23.5% of cases. Immunohistochemistry is the mainstay of diagnosis, mesotheliomas typically test positive for CK5/6, calretinin, WT-1, HBME-1, thrombomodulin, mesothelin, podoplanin (D2–40). No single marker provides 100% specificity; subsequently, ascertain an accurate diagnosis a combination of two or more markers is required [5].

Surgery in the management of PM maybe beneficial to reduce debulk the tumour and relieve symptoms of mass effect. Chemotherapy and concurrent radiotherapy are the mainstay of treatment, extending life-expectancy up to 50-months in some cases [2].

## Conclusion

PMs are a rare and aggressive form of mediastinal tumour tumours. Their presentation is often innocuous and incidental. Due to their rarity, reference imaging is scares and the natural history is poorly understood. Patients may benefit from a combination of surgery, chemotherapy and radiotherapy depending on the extent of disease.

## Conflict of interest statement

The authors have no conflict of interest to declare.

## Funding

None declared.

## Declarations

The patient has provided written consent for this content to be published including images.

Ethics review was conducted by the Gold Coast University Hospital and Health Service Human Research Ethics Committee and granted an exemption from ethics review: EX/2022/QGC/85726.

This project was presented as a poster at the 3sCTS meeting in Cairns, November 2022.
